# Trends in mortality rates from coronary heart disease in Belgrade (Serbia) during the period 1990–2010: a joinpoint regression analysis

**DOI:** 10.1186/1471-2261-13-112

**Published:** 2013-12-09

**Authors:** Isidora S Vujcic, Sandra B Sipetic, Eleonora S Dubljanin, Hristina D Vlajinac

**Affiliations:** 1 Institute of Epidemiology, Faculty of Medicine, University of Belgrade, Visegradska 26, 11000 Belgrade, Serbia; 2 Institute of Microbiology and Immunology, Faculty of Medicine, University of Belgrade, Dr Subotica 1, 11000 Belgrade, Serbia

**Keywords:** Coronary heart disease, Myocardial infarction, Trend, Joinpoint regression, Mortality

## Abstract

**Background:**

Coronary heart disease (CHD) causes an estimated 7 million deaths worldwide each year. In the last few decades, mortality from CHD has been decreasing in many countries. The aim of this study was to analyze the trends of mortality from CHD and myocardial infarction (MI) in the population of Belgrade during the period 1990–2010.

**Methods:**

Mortality data for CHD and MI were obtained from the Municipal Institute of Statistics in Belgrade and used to calculate age- and sex-specific and age-adjusted mortality rates. Joinpoint regression analysis was used to estimate annual percent changes (APCs) in mortality and to identify points in time where significant changes in trend occur.

**Results:**

Trends in CHD mortality rates showed significant decline in men during the period studied (APC -0.5%, no joinpoints detected), but no significant change among women (APC +0.4%, no joinpoints detected). While we observed significant declines in CHD mortality in men aged 35–44, 55–64 and 65–74 and women aged 55–64, there was a significant increase in mortality in men aged ≥85 and women aged 75–84 and ≥85. Trends in MI mortality rates showed similar patterns in both genders, with a significant decline from the mid-1990s. Significant decline in MI mortality was observed in almost all age groups, except the two oldest (75–84 and ≥85) in women population.

**Conclusions:**

Given that CHD and MI mortality trends showed different patterns during the period studied, especially in women, our results imply that further observation of trend is needed.

## Background

Cardiovascular diseases (CVDs) are the leading cause of death worldwide, accounting for 17.3 million deaths in 2008 and 30% of total global mortality [1]. Coronary heart diseases (CHDs) are responsible for a further estimated 7 million deaths annually, accounting for 13% of all male and 12% of all female deaths [2]. It is estimated that the number of deaths from CHD and stroke will increase to 23.3 million by 2030, remaining the leading causes of death worldwide [1].

Mortality rates from CHDs have been declining in the United States of America, Australia and Northern and Western Europe, with approximately two thirds of the decline attributable to changes in risk factors and one third due to evidence-based treatments [3-5]. Meanwhile, in other countries, such as former Soviet Republics and other Eastern European countries, mortality rates have continued to increase. This is in contrast with Japan and several Southern European countries, where mortality has remained relatively low and stable over time [3]. Significant declines in mortality due to CVD and CHD have been observed over the past few decades across the European Union, with rates of CVD mortality falling by 30% in men and women, and CHD mortality falling by third in men and over a quarter in women from 1985–1989 to 2000–2004 [6]. In the past few years, developed countries have reported that mortality rates of CHD have stopped decreasing in younger age groups, and may even have increased for the first time in over two decades [7,8]. CHD mortality trends in Serbia’s neighboring countries are generally unfavorable in comparison with trends in Northern, Western and Southern European countries, showing for example non-significant increase in Croatia during the period 1997–2006 by 0.6% annually, for persons over 18 years [9] and significant increase in Romanian men during the last three decades by 1.0% annually [10]. On the contrary, in Bulgaria there was significant decline during the period 1980–2009 (although in the first decade there was significant increase). Taking into account big political and economic changes in Serbia from 1990 and onward and their possible impact on population health, the aim of this descriptive epidemiological study was to analyze trends in mortality from CHD and myocardial infarction (MI) in Belgrade during the twenty-one-year period from 1990 to 2010.

## Methods

Mortality data for CHD (International Classification of Diseases ICD-9 codes 410–414 & ICD-10 codes I20–I25) and MI (ICD-9 code 410 & ICD-10 codes I21–I22) were obtained from the Municipal Institute of Statistics in Belgrade. Deaths from MI were included in the numerator for deaths from CHD. We used projected Belgrade population figures, which were extracted from unpublished data from the Statistical Office of the Republic of Serbia, as the denominator for each year. We calculated age- and sex-specific mortality rates for CHD and MI, and then standardize them using the direct method, according to the European Standard population [11]. Age-specific mortality rates were calculated by 10 years age groups for persons aged 35 years and older, and those aged ≥85 formed the last open-ended age group. Trends in mortality rates were evaluated using joinpoint regression analysis (Joinpoint Regression Program, Version 4.0.4 May 2013; Statistical Methodology and Applications Branch, Surveillance Research Program, National Cancer Institute), according to the method proposed by Kim et al. [12]. Joinpoint regression was fitted to estimate average annual percent changes (APCs) and identify points in time at which significant changes in trends occurred. A maximum of three joinpoints was allowed for each estimation, and for each segment APC was computed using a log-linear model. In addition, 95% confidence intervals were calculated for each estimate of APC and were used to determine if the APC for each segment differed significantly from zero. The study was approved by the Ethics Committee of Medical Faculty, University of Belgrade (number 440/IX-2).

## Results

During the observed period of twenty-one-years 1048 men and 765 women died from coronary heart diseases annually. The average age-adjusted CHD mortality rate was higher in men (127.8 per 100,000) than in women (68.4 per 100,000). For MI, average age-adjusted mortality rates were 72.6 per 100,000 in men and 45.9 per 100,000 in women. Proportional contribution of MI to CHD mortality was approximately 75% in men, and 68% in women, although this had been declining in recent years, especially in women (Table 1).

**Table 1:** Number of deaths, crude and age-adjusted (European population) coronary heart disease and myocardial infarction mortality rates per 100,000 for men and women, Belgrade 1990–2010

**Years**	**Men**						**Women**					
	**CHD**			**MI**			**CHD**			**MI**		
	**No**	**Crude rates**	**AARs**	**No**	**Crude rates**	**AARs**	**No**	**Crude rates**	**AARs**	**No**	**Crude rates**	**AARs**
1990	898	120.3	131.6	613	82.2	87.7	547	67.8	63.3	350	43.4	39.4
1991	995	133.3	142.9	676	90.6	95.4	596	73.9	69.5	359	44.5	40.6
1992	1005	134.2	142.9	770	102.8	106.0	545	67.2	62.7	390	48.1	43.6
1993	910	121.1	126.6	792	105.4	108.7	570	70.0	61.7	461	56.6	49.6
1994	912	121.0	123.5	778	103.2	104.0	620	75.9	66.5	482	59.0	50.8
1995	971	128.4	127.7	821	108.6	105.8	653	79.6	67.2	521	63.5	53.1
1996	1056	139.4	136.1	870	114.9	111.4	762	92.6	76.1	630	76.6	62.3
1997	1129	149.5	144.5	929	123.0	116.3	766	93.1	74.6	580	70.5	56.0
1998	1102	146.8	137.5	888	118.3	109.8	776	94.3	73.5	576	70.0	54.0
1999	997	133.5	120.5	821	109.9	98.2	728	88.6	66.5	524	63.8	47.6
2000	995	133.6	118.7	791	106.2	92.7	725	88.3	64.5	532	64.8	47.1
2001	979	131.3	116.7	767	102.9	90.4	704	85.4	62.4	524	63.5	45.8
2002	1019	136.1	121.7	833	111.2	99.0	777	93.7	69.2	589	71.0	51.7
2003	1054	140.6	127.0	854	113.9	103.0	810	97.4	71.1	625	75.1	54.1
2004	966	128.3	109.3	700	93.0	78.4	674	80.6	55.5	438	52.4	35.8
2005	1058	140.0	118.9	720	95.3	79.5	791	94.1	64.5	484	57.6	38.8
2006	1297	171.1	140.9	845	111.5	90.8	1024	121.2	78.7	535	63.3	42.1
2007	1158	152.1	126.8	729	95.7	78.9	1029	121.1	77.3	560	65.9	41.7
2008	1194	155.9	127.3	776	101.3	82.3	980	114.5	72.3	549	64.2	40.7
2009	1178	153.1	123.8	750	97.5	78.5	998	115.9	70.3	495	57.5	35.4
2010	1143	147.9	118.1	699	90.4	72.6	986	113.8	69.1	490	56.6	34.7
Average	1048	138.9	127.8	782	90.4	72.6	765	91.8	68.4	509	61.3	45.9

CHD: coronary heart diseases, MI: myocardial infarction, AAR: age-adjusted rate.

**Figure 1:**
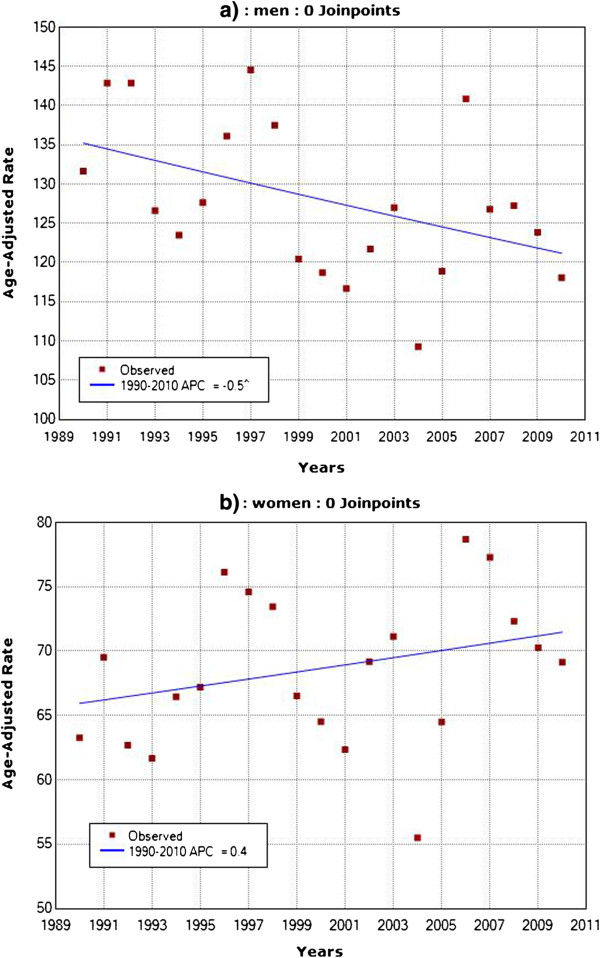
Observed and estimated trends in age-adjusted coronary heart disease mortality rates in a) men and b) women in Belgrade, 1990–2010.

Trends in age-adjusted mortality rates for CHD showed significant decrease during the period 1990–2010 in men (APC -0.5%, no joinpoints detected), and non-significant change in women, with a small, steady increase (APC +0.4%, no joinpoints detected) (Figure 1).

**Figure 2:**
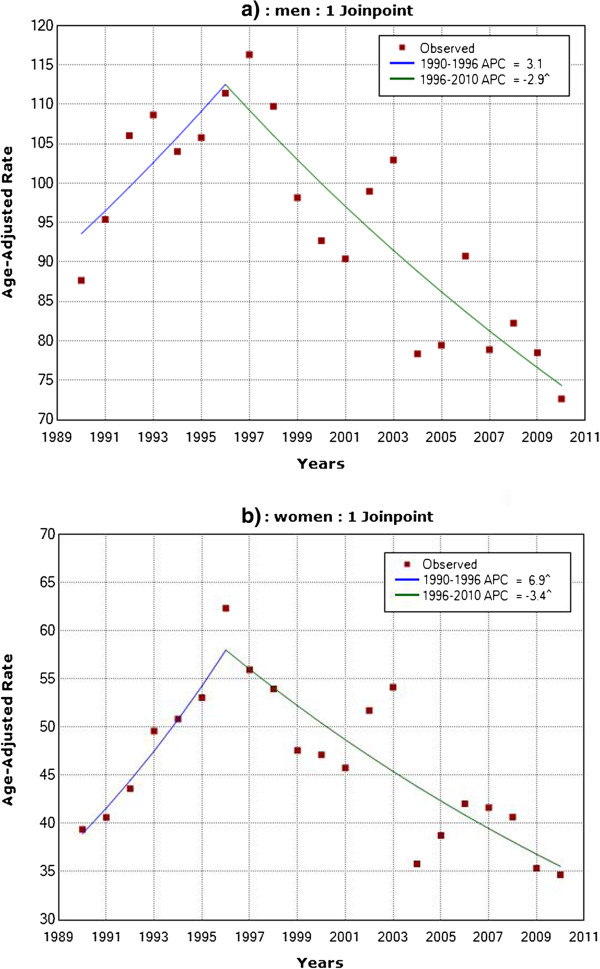
Observed and estimated trends in age-adjusted myocardial infarction mortality rates in a) men and b) women in Belgrade, 1990–2010.

Trends in age-adjusted mortality rates for MI showed similar pattern in men and women. One joinpoint was detected for men, with a non-significant increase in the MI mortality trend from 1990–1996 (APC + 3.1%; 95% CI: -0.6 to 7.0) and a significant decrease from 1996–2010 (APC -2.9%; 95% CI: -3.8 to -2.0). In women, one joinpoint was also found, showing a significant increase from 1990 to 1996 (APC +6.9%; 95% CI: 2.0 to 11.9) and a significant decrease from 1996 to 2010 (APC -3.4%; 95% CI: -4.5 to -2.3) (Figure 2).

A significant increase in mortality from CHD was observed in men aged ≥85 (APC +1.7%, no joinpoints detected), and a non-significant increase in men aged 75–84. In all other age groups, however, we observed a decline in CHD mortality over the entire period studied, which was significant for men aged 35–44, 55–64 and 65–74 (-1.5%, -1.7% and -1.1% respectively) and non-significant for men aged 45–54 (Table 2). For women we found a statistically significant rising trend in CHD mortality in the two oldest age groups (75–84 and ≥85) for the entire period studied (APC +1.2% and +3.3%, respectively), while for women aged 55–64 significant decrease in mortality was observed (APC -2.5%, no joinpoints detected). There was also a non-significant decrease of CHD mortality among women aged 35–44 and 65–74 after the mid-1990s and women aged 45–54 over the entire period studied.

**Table 2:** Joinpoint analysis: trends in age-specific coronary heart disease mortality rates, men and women, Belgrade, 1990–2010

**Age**	**Period**	**APC**	**Lower 95% CI**	**Upper 95% CI**
Men				
35–44	1990–2010	-1.5*	-2.9	-0.1
45–54	1990–2010	-0.5	-1.2	0.2
55–64	1990–2010	-1.7*	-2.2	-1.2
65–74	1990–2010	-1.1*	-1.9	-0.3
75–84	1990–2010	0.2	-0.7	1.1
≥85	1990–2010	1.7*	0.5	2.8
Women				
35–44	1990–1995	29.4	-0.2	67.7
	1995–2010	-3.7	-7.8	0.6
45–54	1990–2000	-1.2	-2.8	0.3
55–64	1990–2010	-2.5*	-3.2	-1.8
65–74	1990–1996	9.0*	4.4	13.7
	1996–2001	-5.4	-11.3	0.8
	2001-2010	-0.7	-2.7	1.4
75–84	1990–2010	1.2*	0.1	2.3
≥85	1990–2010	3.3*	2.1	4.6

CI: confidence interval, APC: annual percent change, *Significantly different from 0.

Declines in mortality rates from MI were identified in almost all age groups, except in two oldest age groups in women where a non-significant increase was present. Significant declining trends in MI mortality were observed for men aged 35–44 and 75–84 for the entire period. This was also the case for: those aged 45–54 years from 1993, those aged 55–64 from 1995, and those aged 65–74 from 1996. For women, significant decrease in MI mortality started in 1998 and 1996 for those aged 55–64 and 65–74 years respectively, while for those aged 45–54 years it was present over the entire period studied (Table 3).

**Table 3:** Joinpoint analysis: trends in age-specific myocardial infarction mortality rates, men and women, Belgrade, 1990–2010

**Age**	**Period**	**APC**	**Lower 95% CI**	**Upper 95% CI**
Men				
35–44	1990–2010	-2.0*	-3.5	-0.4
45–54	1990–1993	11.8	-4.7	31.3
	1993–2010	-1.5*	-2.5	-0.5
55–64	1990–1995	3.6	-1.4	8.9
	1995–2010	-3.2*	-4.2	-2.2
65–74	1990–1996	4.2*	-0.5	9.2
	1996–2010	-3.7*	-4.9	-2.6
75–84	1990–2010	-1.2*	-2.1	-0.2
≥85	1990–2010	-1.6	-3.8	0.5
Women				
35–44	1990–1995	27.8	-4.6	71.2
	1995–2010	-3.6	-8.2	1.3
45–54	1990–2010	-1.7*	-3.1	-0.2
55–64	1990–1998	0.0	-3.1	3.3
	1998–2010	-5.4*	-7.2	-3.4
65–74	1990–1996	11.7*	7.1	16.5
	1996–2010	-5.3*	-6.3	-4.3
75–84	1990–2010	0.9	-2.0	0.3
≥85	1990–2010	1.0	-0.9	3.0

CI: confidence interval, APC: annual percent change, *Significantly different from 0.

## Discussion

Our results indicate a discrepancy between the trends in CHD an MI mortality rates, especially in women. In the twenty-one-year period from 1990 to 2010, MI mortality rates decreased significantly after the mid-1990s in both genders, in contrast with the significant increase observed in the early-1990s, which was significant in women but non-significant in men. CHD mortality rates showed significant decrease in men, and no significant change in women with a slow, steady increase over the period studied. In their analysis of trends in CHD mortality across 26 European Union member states, Nichols et al. found significant falls in mortality rates among both men and women over the last three decades in almost all countries [10]. The average APC in mortality for all age groups between 1980 and 2009 was -2.7% for men and -2.4% for women. The largest decreases in CHD mortality rates for men in Europe were observed in Denmark, Malta, the Netherlands, Sweden and the United Kingdom. Trend similar to this in Serbia was noticed in Slovakia - significant decline (APC -0.6%) during the whole period, but small in comparison with other countries. Non-significant decreases in mortality rates were observed among men in Hungary, Latvia, Lithuania and Poland. The study also found non-significant changes in mortality rates for women in Greece, Hungary, Lithuania, Poland, Romania and Slovakia. Dinc et al. [13] studied CHD mortality trend in Turkey from 1988 to 2008 and found that mortality rates increased by 2.9% in men and 2.0% in women annually from 1988 to 1994, and then started to decline. The annual decline of rate for men was 1.7% between 1994–2008, while in women it was 2.8% between 1994–2000 and 6.7% between 2005–2008. Our results indicated a decrease in MI mortality rates, with an APC of -2.9% for men and -3.4% for women after the mid-1990s.

During the period 1990–2010 the average age-adjusted CHD mortality rates in Belgrade population were 127.8 per 100,000 in men and 68.4 per 100,000 in women. In order to make comparison with European countries age-adjusted CHD mortality rates for persons aged 45–74 years were also calculated (Additional file 1: Table S1). In the year 2000 they were 246 per 100,000 in men and 124 per 100,000 in women. Similar rates to those in Serbia were observed for men in Germany, Sweden and Greece, and for women in Poland and Northern Ireland [14]. In our neighboring countries rates per 100,000 were much higher in both men (Macedonia 328, Croatia 381, Bulgaria 404, Romania 449, Hungary 529) and women (Croatia 136, Macedonia 145, Bulgaria 157, Hungary 202 and Romania 209). The age-adjusted MI mortality rates, for persons aged 45–74 years, were 205 per 100,000 in men and 91 per 100,000 in women in the year 2000.

CHD mortality rates have shown a decrease since the late 1970s in most Western European countries, and since early 1970s in North America. This decrease was greater in some countries such as Finland and the Netherlands when compared with others (Germany, Ireland, and Portugal), and started later in countries such as Denmark and Germany [15]. In developed countries such as Australia, the United States of America, the United Kingdom and the Netherlands the decline in CHD mortality rates is now slowing among young adults under 55 [7,8,16,17]. Trends varied among Eastern European countries, although they were generally unfavorable especially until the late 1990s [15]. Most of these countries, including Serbia’s neighbors (Croatia, Romania and Bulgaria), and especially Russia, showed persisting upward trends in CHD mortality rates. Exceptions include Poland and the Czech Republic, where rates increased up to the mid-1990s and then decreased significantly in the subsequent period. In Hungary, Serbia’s northern neighbor, mortality rates stabilized at a high level in the mid-1990s. In some former Soviet Republics upward trends in CHD mortality are present even nowadays [18].

Our results, which show a significant decrease in MI mortality rates and no change in CHD mortality trend in women, may suggest that mortality rates from other CHDs such as angina pectoris, complications following acute MI and other acute CHDs and chronic CHDs may have increased. According to data from the Serbian Registry for acute coronary disease for the year 2011, CHD was the third-most frequent cause of death from CVDs [19]. While other heart diseases were cited as the most common cause of death, accounting for 41.7% of all-cause mortality, cerebrovascular diseases and CHD accounted for 29.4% and 22.6% of deaths, respectively. While nearly half of all CVD deaths in Europe in both men and women were a result of CHD, this figure is even greater in the United States of America [18,20]. In addition, registry data from 2006 to 2010 showed that on average 56.2% (range 54.2%-60.8%) of all CHD deaths in Serbia were due to acute coronary syndrome (whose definition encompasses unstable angina, acute and recurrent MI), while other CHDs accounted for an average of 43.8%. These differences in CHD mortality rates between Serbia and the rest of Europe may indicate the reporting errors and the poor overall quality of data collection. It may also indicate systemic problems in the coding of death certificates, such as the use of ill-defined codes for CVDs. There are a number of codes in the ICD 9 and 10 (heart failure, ventricular dysrhythmias, generalized atherosclerosis and ill-defined descriptions and complications of heart disease) under which physicians may incorrectly assign deaths that are actually due to CHD [21]. However, these coding errors are unlikely to have affected MI mortality estimates given that the MI death codes are clearly defined and can be verified objectively [22]. The change from ICD-9 to ICD-10 may also be another potential cause for coding errors. Griffiths et al. examined the impact of the introduction of ICD-10 on mortality from circulatory diseases in England and Wales, and found that trends in mortality from CHD were unaffected, but number of MI deaths decreased by around 10% with this coding change [23]. Taking this into account it could be presumed that the introduction of ICD-10 was at least partly responsible for decline of MI mortality in Belgrade population. The Serbian Register for acute coronary syndromes, which was established in 2006 to facilitate the monitoring of incidence rates and mortality for selected diseases, may contribute to improvement of data collection in the future. In recent years, while the percentage of other heart diseases has been decreasing as a proportion of all CVDs, the relative proportion of CHD cases has increased, indicating that improvements in coding causes of death have already taken place. Although the discrepancy between the trends in CHD and MI was much less present in man, it was still visible at the beginning of the study period.

In developed countries, CHD mortality rates decreased in all age groups, including the oldest [24,25]. This was not the case in Serbia, where significant increases were observed in the oldest age groups both in men and women. This postponement of CHD mortality could result from reductions in exposure to risk factors, which may have contributed to the age gradient identified by the present study [26]. In Serbia, the prevalence of risk factors such as hypertension, diabetes mellitus, and hyperlipidemia is significantly higher in those over the age of 45 compared with younger people [27]. It is also possible that younger age groups have enjoyed improved access to medication and surgical treatment, which may have led to a reduction of mortality in this group [26]. In addition, higher reported rates of mortality may be a result of improved registration and reporting, especially for women, among whom the characteristic symptoms of CHD and MI are often not apparent.

The significant rising trend in MI until the mid-1990s can be explained by the difficult political and economic situation faced by the people of Serbia, including the most severe hyperinflation recorded globally during the mid-1990s. This situation resulted from the collapse of the Socialist Federal Republic of Yugoslavia and the refugee crisis precipitated by the outbreak of war in the region.

Subsequent decreasing trend of MI mortality, present in almost all age groups in both genders, may be the result of reduced exposure to risk factors (for example, the smoking prevalence in Serbia was reduced by 6.8% during the period 2000–2006) [27] including better treatment of hypertension, hyperlipidemia, diabetes mellitus and also improved treatment and survival of MI patients.

## Conclusion

Rates of mortality for CHD and MI showed different patterns of change during the period 1990–2010, especially in women, implying that further analysis of trend is needed.

## Abbreviations

CVD: Cardiovascular disease; CHD: Coronary heart disease; MI: Myocardial infarction; APC: Annual percentage change; AAR: Age-adjusted rate; CI: Confidence interval.

## Competing interests

The authors declare that they have no competing of interests.

## Authors’ contributions

IV - conceived and designed study, acquired, analyzed and interpreted data, and wrote manuscript. SS – participated in plan of the study and revised manuscript for important intellectual content. ED - participated in the design and helped to draft the manuscript and wrote some parts. HV – revised manuscript critically for important intellectual content and gave final approval of the version to be published. All authors read and approved the final manuscript.

## Pre-publication history

The pre-publication history for this paper can be accessed here:

http://www.biomedcentral.com/1471-2261/13/112/prepub

## Supplementary Material

Additional file 1: Table S1 Age-adjusted coronary heart diseases and myocardial infarction mortality rates for men and women aged 45-75 years, Belgrade, 1990-2010. Click here for file
