# Supplementary material for: Trends in mortality rates from coronary heart disease in Belgrade (Serbia) during the period 1990–2010: a joinpoint regression analysis
Source: BMC Cardiovasc Disord. 2013 Dec 9;13:112. doi: 10.1186/1471-2261-13-112 (PMC3878899; doi:10.1186/1471-2261-13-112)
Supplement: Additional file 1: Table S1 — Age-adjusted coronary heart diseases and myocardial infarction mortality rates for men and women aged 45-75 years, Belgrade, 1990-2010. [file 1471-2261-13-112-S1.pdf]

Additional file

Table S1. Age-adjusted coronary heart diseases and myocardial infarction mortality rates for men and women ages 45-75 years, Belgrade, 1990-2010.

	Age-adjusted (European population) coronary heart diseases mortality rates per 100,000		Age-adjusted (European population) myocardial infarction mortality rates per 100,000	
Years	Men	Women	Men	Women
1990	246.9	107.6	174.8	67.8
1991	281.3	123.5	196.4	74.5
1992	280.9	109.9	223.8	78.8
1993	254.3	129.5	225.2	96.7
1994	247.5	135.8	215.0	97.2
1995	264.7	132.3	229.3	98.5
1996	271.5	131.6	224.8	106.3
1997	284.1	138.9	245.5	95.9
1998	271.5	137.7	224.1	96.4
1999	243.4	119.3	207.6	85.9
2000	245.7	123.7	205.1	90.6
2001	229.4	106.8	186.9	81.5
2002	229.0	108.3	192.4	82.2
2003	227.0	111.3	187.5	80.9
2004	219.0	104.2	165.7	63.9
2005	229.2	112.0	164.1	66.1
2006	257.0	112.5	179.7	67.0

2007	227.7	108.8	162.4	63.2
2008	228.5	106.7	166.1	63.0
2009	215.1	95.8	155.4	51.7
2010	206.7	102.1	149.5	52.8
Average	245.7	117.1	194.4	79.1
